# Utilization Patterns Among Heterogeneous Subgroups of Homebound Older Adults: A Latent Class Analysis

**DOI:** 10.1111/jgs.70576

**Published:** 2026-07-03

**Authors:** J. Lee Young, Mary L. Pomeroy, Hanna Charankevich, Katherine A. Ornstein, Bruce P. Kinosian

**Affiliations:** ^1^ Department of Medicine, Division of Geriatrics Perelman School of Medicine, University of Pennsylvania Philadelphia Pennsylvania USA; ^2^ Center for Equity in Aging, School of Nursing, Johns Hopkins University Baltimore Maryland USA

**Keywords:** frailty, healthcare utilization, home‐based care, latent class analysis

## Abstract

**Background:**

Homebound older adults represent a high‐risk, high‐needs population characterized by significant clinical and sociodemographic heterogeneity. Little is known about how such heterogeneity shapes care needs and utilization patterns. We characterized utilization patterns among distinct subgroups of homebound older adults and examined associations between levels of outpatient care and emergency department (ED) utilization.

**Participants and Setting:**

We used nationally representative data from the National Health and Aging Trends Study (NHATS) linked to Medicare Fee‐For‐Service (FFS) claims from 2011 to 2019 and 2021–2023 to examine trends among homebound older adults ages 70 or older.

**Methods:**

In this retrospective observational study, we characterized utilization patterns among distinct subgroups of homebound older adults identified using latent class analysis (LCA). We then used multivariable regression to examine associations between level of outpatient utilization and probability of both any ED visit and any potentially avoidable ED visit identified using Ambulatory Care Sensitive Conditions (ACSC). We adjusted for other indicators of ED use and access to care (i.e., self‐reported health, rurality, and disease burden).

**Results:**

There was a total of 2202 person‐year observations across 1343 unique homebound individuals. The LCA identified four subgroups of homebound older adults: functional independence; dementia; multimorbidity; and dementia plus multimorbidity. There were significant differences in levels of ambulatory and acute care utilization between subgroups. Regression analyses did not show evidence to support the conventional wisdom that inability to access ambulatory care leads to more avoidable ED visits among the homebound older adult population.

**Conclusions:**

Heterogeneity among the homebound older adult population shapes care needs that in turn influence utilization patterns. Understanding these dynamics is crucial to targeting interventions like home‐based primary care to the highest risk groups while tailoring care to individual needs.

## Summary

- Key points
  ○ In this nationally representative study of homebound Medicare beneficiaries not receiving home‐based primary care (HBPC), we identified four distinct subgroups of homebound older adults characterized by: functional independence, dementia, multimorbidity, and dementia plus multimorbidity.
  ○ Distinct subgroups of homebound older adults have unique care needs that shape differences in care utilization, but we did not find any evidence that homebound status necessarily connotes an inability to access outpatient care leading to higher rates of avoidable ED use.
  ○ Further research is needed to understand how alternative care delivery models such as HBPC impact utilization patterns differently among distinct subgroups of homebound older adults.
- Why does this paper matter?
  ○ Homebound older adults represent a uniquely high‐risk, high‐needs population characterized by overall greater health care utilization and costs than their non‐homebound peers. Significant heterogeneity exists among the homebound in terms of clinical and sociodemographic characteristics, but little is known about how such heterogeneity impacts utilization patterns.
  ○ This study contributes to efforts to better understand how heterogeneity among the homebound shapes distinct care needs, differential barriers to access, and patterns of utilization. Such insights are crucial for developing interventions that effectively target the highest‐risk groups while tailoring care to individual care needs.

## 1 Introduction

An estimated two million community‐dwelling older adults in the United States are homebound, defined as leaving the home once per week or less [1]. The homebound comprise a high‐risk, high‐needs population, with grater prevalence of multimorbidity, functional impairment, social isolation, frailty, dementia, and depression when compared to the non‐homebound [1, 2, 3, 4, 5]. They are more likely to be low‐income, have less educational attainment, and belong to minority racialized groups [1]. At the same time, there is significant heterogeneity among the homebound. A recent study using Latent Class Analysis (LCA) identified four subgroups characterized by unique clinical and sociodemographic profiles among a nationally representative sample of newly homebound older adults [6]. The authors noted that distinct health and functional status profiles likely indicate different underlying care needs, but little is known about how such differences influence health care utilization patterns.

Homebound older adults have higher rates of emergency department (ED) visits, hospitalizations, and skilled nursing facility stays than the non‐homebound, but receive less routine primary and outpatient specialty care [1, 2, 7, 8, 9, 10]. These trends generate higher costs among this group [9].

In addition to greater levels of medical and social complexity, access barriers inherent to being homebound, such as immobility and difficulty traveling to clinic, are frequently cited as factors limiting access to routine care and contributing to avoidable acute care use, especially avoidable ED visits [11, 12, 13, 14, 15, 16, 17]. To address this conventional wisdom of outpatient care inaccessibility among the homebound, a growing array of home‐based primary care (HBPC) models are designed to overcome such barriers to deliver effective care while curbing overall costs. Unfortunately, access to such programs remains limited, with only a small minority of homebound older adults receiving these services [1]. In this context, understanding the heterogeneity of the homebound, including how distinct care needs and differential barriers impact utilization, can inform strategies for targeting the highest risk groups while tailoring care to individual needs.

The aims of this retrospective observational study are threefold. First, we use data from the National Health and Aging Trends Study (NHATS) to determine the existence of distinct subgroups among a nationally representative *prevalent* sample of homebound older adults enrolled in Traditional Medicare and not receiving HBPC. Second, we characterize utilization patterns among subgroups. Finally, we examine associations between levels of outpatient care and ED utilization and whether these differ between subgroups. Specifically, we assessed the assumption that lack of outpatient care among the homebound leads to greater ED use by determining whether lower rates of outpatient care were associated with a higher likelihood of ED utilization, including potentially avoidable ED visits.

## 2 Methods

### 2.1 Data Source and Sample

We used data from NHATS linked to Medicare Fee‐For‐Service (FFS) claims between 2011–2019 and 2021–2023. NHATS is a longitudinal study of late‐life disability trends using annual interviews among a nationally representative sample of Medicare beneficiaries 65 years and older. Participants and/or proxy respondents complete interviews addressing health and functional status and socioeconomic characteristics [18]. We constructed a prevalence sample of homebound FFS‐enrolled participants aged 70 years and older with linked Medicare claims data in weighted person‐years using NHATS sampling and weighting guidelines [18]. We determined homebound status using responses to an NHATS item regarding ability to leave the home in the 30‐day period prior to the interview. We followed NHATS‐defined criteria of never or rarely (once per week or less) leaving the home over the past 30 days to classify homebound status [1].

We excluded data from 2020 to account for COVID pandemic‐related rises in homebound status [19]. We used Medicare claims data to identify and exclude individuals enrolled in HBPC because these programs are designed to mitigate barriers to outpatient care related to being homebound. We excluded individuals residing in nursing homes and those not enrolled in FFS Medicare or with missing claims data.

### 2.2 Measures

#### 2.2.1 LCA Measures

Measures of health, functional status, sociodemographic characteristics, and geographic and caregiving contexts were taken from the index NHATS interview in which homebound status was determined for each person‐year observation. Continuous variables were converted to categorical values. We drew LCA indicator variables from Mather et al. [6], who based selection on prior literature and clinical experience pertaining to sociodemographic and clinical characteristics associated with homebound status.

##### 2.2.1.1 Sociodemographics

Included age (< 85 vs. ≥ 85), sex (male vs. female); race and ethnicity (White non‐Hispanic, Black non‐Hispanic, Hispanic, other); marital status (married vs. all else); education level (high school or above vs. less than high school), income level (< 100% federal poverty line vs. all else); Medicaid status and financial strain [20, 21] (self‐report of lacking money to pay rent or mortgage, utilities, or medication/prescription bills, or skipping meals due to inability to afford food).

##### 2.2.1.2 Health and Functional Status

Included measures of frailty (using physical frailty phenotype [22], defined as having any three of the following: weight loss, exhaustion, slow walking speed, low physical activity, low grip strength); functional impairment (dependence in ≥ 2 activities of daily living [ADLs] by self‐report of requiring help with the activity in the prior month); self‐rated health (dichotomized as fair or poor vs. good, very good or excellent); mobility impairment (self‐report of inability to walk 6 blocks); sensory impairment (self‐report of vision and/or hearing difficulty); probable dementia (based on NHATS validated criteria [23] including self‐report of dementia, proxy responses to the Alzheimer's Disease (AD)‐8 screening tool, and cognitive testing in memory, orientation and executive function domains); depressive symptoms (defined as scoring ≥ 1 on PHQ‐2 screening questions); self‐reported activity‐limiting pain; self‐reported breathing problems; self‐reported hospitalization in the past year; and self‐reported falls in the past month; self‐report of any of the following chronic conditions: heart attack, heart disease, high blood pressure, stroke, lung disease, cancer, osteoporosis, hip fracture, arthritis, anxiety, which was used to construct a multimorbidity variable defined as ≥ 3 of any chronic diseases.

##### 2.2.1.3 Caregiving Context

Included residing in assisted living, living alone, presence of paid caregiver, and caregiver network size.

##### 2.2.1.4 Geographic Characteristics

Sorted by census region based on county of residence.

#### 2.2.2 Outcome Measures

All utilization data were derived from NHATS‐linked Medicare Parts A and B claims. Claims data for each person‐year observation were collected for the 12 months following the index NHATS interview. Outcomes included: hospital admissions; ED visits; avoidable ED visits; primary care visits; and outpatient specialty care visits. Primary and specialty telehealth visits were included. Healthcare Common Procedure Coding System and Provider Specialty codes were used to identify primary and specialty care visits. ICD codes associated with ED visits were used to identify Ambulatory Care Sensitive Conditions (ACSCs) as an indicator of potentially avoidable ED visits. ICD‐10 diagnostic codes for acute ACSCs were taken from the Agency for Healthcare Research and Quality (AHRQ) Prevention Quality Indicator (PQI) for ED settings version 2025 [24]. Chronic ACSC diagnostic codes were taken from AHRQ PQI 90 version 2025 [25].

In regressions, outpatient care was the independent variable and included all primary and specialty outpatient care including telehealth. Levels of outpatient care were classified as “none” (0 visits per year), “low” (1–4 visits), or “high” (5+ visits). Cut‐offs were based on empirical distributions that divided the population into tertiles. ED utilization was the dependent variable and was coded as a binary outcome (ED visit vs. none). In the first set of analyses, all ED visits were included. In subsequent analyses, only ACSC‐related ED visits were included.

#### 2.2.3 Covariates

Self‐reported health and disease burden were used as proxies for subjective and objective care needs, respectively. Self‐reported health was taken directly from NHATS surveys. We used ICD codes from claims data to calculate Hierarchical Conditions Categories (HCC) for each person‐year observation. We used NHATS‐derived personal address data to calculate Rural–Urban Community Area (RUCA) scores to adjust for geography‐based differences in access to inpatient and outpatient care.

### 2.3 Analyses

We performed LCA to identify distinct latent classes of homebound older adults within our prevalence sample using a similar approach to Mather et al. [6] in their incident sample. One‐ to five‐class models were compared for goodness of fit using Akaike Information Criterion and Bayesian Information Criterion indices. All indicator variables were taken from the NHATS‐derived measures described above. After model selection, each observation was assigned to one class based on posterior probabilities. Characteristics of member observations were then used to name respective classes.

We calculated unadjusted weighted means among the total sample and for each subgroup identified by LCA for the following utilization categories: any ED visit; any hospital admission; any avoidable ED presentation; any primary care visit; any specialty care visit; and any primary or specialty care visit.

Multivariable logistic regression examined associations between levels of outpatient care utilization and likelihood of ED utilization. Analyses were repeated for each subgroup identified through the LCA. We adjusted for NHATS‐derived self‐reported health, rurality based on RUCA scores, and disease burden using HCC scores. The “none” outpatient care group was the reference group in regression analyses. We examined associations for all ED visits and ACSC‐related visits. We further stratified by acute and chronic ACSCs. Sensitivity analyses excluded outpatient visits occurring within 2 and 4 weeks of any ED visit to exclude post‐discharge follow‐up visits.

## 3 Results

### 3.1 Baseline Population Characteristics

There were a total of 2202 person‐year observations representing 1343 unique homebound individuals with Medicare FFS claims‐linked data. Descriptive characteristics are included in Table 1. Average age was 83.4 and most were female (73.3%) and living in urban settings (80.2%). A majority were White non‐Hispanic (74.2%), followed by Hispanic (12.9%), Black non‐Hispanic (9.4%), and “Other” (3.4%). About half were in the lowest income quartile (46.2%) and a third were living below the poverty line (35.8%) and receiving Medicaid (30.4%). Over half were frail (58.7%) and/or dependent in 2+ ADLs (53.9%), while two‐thirds had ≥ 3 comorbidities (67.4%). The overwhelming majority had limited mobility (90.9%). Depressive symptoms (40.0%) and dementia (43.5%) were prevalent, as were activity‐limiting pain (47.9%) and hospitalization within the previous year (44.2%).

**TABLE 1:** Demographic characteristics.

Characteristic	Homebound enrolled in TM (*n* = 2202)	
	Weighted mean or %	Count
Age	83.4	
Female	73.3	1657
Education ≥ high school	67.1	1377
Lowest income quartile	46.2	1108
Below poverty line	35.8	877
Speaks language other than english	20.5	479
Married	28.3	532
Assisted living facility	17.9	323
Lives alone	42.2	895
Medicaid beneficiary	30.4	806
Frailty	58.7	1369
Probable dementia	43.5	1083
≥ 3 comorbidities	67.4	1450
Depression symptoms by PHQ2	40.0	846
Heart disease	5.8	448
Lung disease	29.7	617
Activity limiting pain	47.9	1054
Dyspnea	35.8	745
Fall in past month	22.3	446
Hospitalization in past 12 months	44.3	983
Self‐report of fair or poor health	59.1	1286
Lives in metropolitan area	80.2	1755
Mobility impaired (Can Ambulate < 6 Blocks)	90.9	2047
White, non‐Hispanic	74.2	1406
Black, non‐Hispanic	9.4	557
Other	3.4	95
Hispanic	12.9	261
No sensory impairment	62.7	1391
Hearing impairment only	21.1	548
Visual impairment only	10.0	338
Dual sensory impairment	6.2	270
Receives help with 0 ADLs	31.3	626
Receives help with 1 ADLs	14.8	334
Receives help with 2+ ADLs	53.9	1242
Northeast	15.0	304
Midwest	19.7	466
South	45.2	1065
West	20.1	367

Abbreviations: ADL, activities of daily living; PHQ2, Patient Health Questionnaire‐2; TM, traditional medicare.

### 3.2 LCA

**FIGURE 1:**
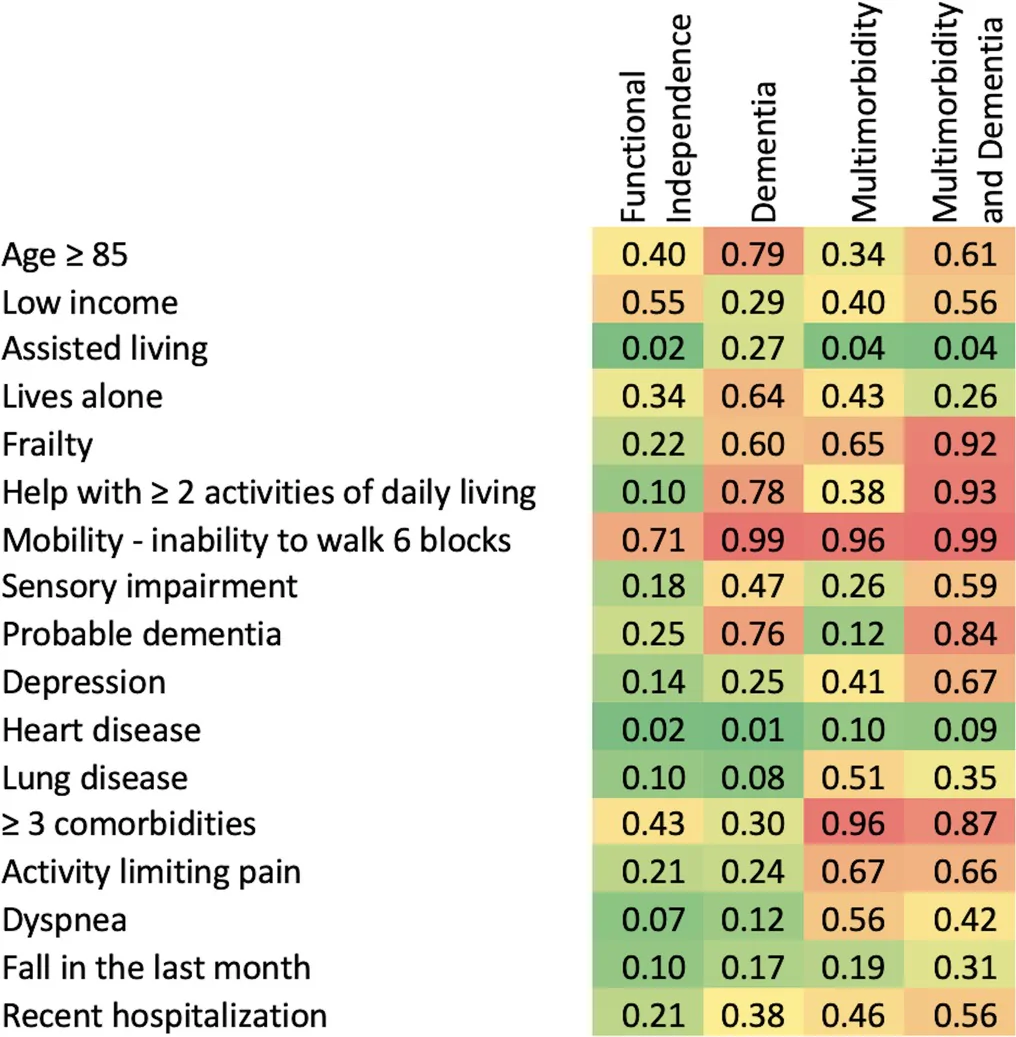
Heat map of estimated margins of each criterion per latent class among prevalent homebound sample in NHATS 2011–2019, 2021–2023; color gradient indicates the probability of a given characteristic conditional on class membership.

Model fit indices supported a 4‐class solution for the LCA. The four subgroups are captured in the Figure 1 heat map and were characterized as follows:

#### 3.2.1 Class 1: Functional Independence (*n* = 930, 18.7%)

The smallest subgroup was characterized by a lower probability of functional impairment in ≥ 2 ADLs (10%). Members had the lowest probability of having frailty (22%), recent fall (10%), or hospitalization in the past year (21%), and less likelihood of activity‐limiting pain, dyspnea, multimorbidity, depressive symptoms, or sensory impairment. A large majority (71%) exhibited an inability to walk six blocks, though overall this group had the lowest probability of mobility impairment. Over half were low‐income.

#### 3.2.2 Class 2: Dementia (*n* = 1394, 28.1%)

Seventy‐six percent of members of this subgroup had probable dementia. They also had the highest probability of being over 85 (79%) and the lowest probability of having ≥ 3 comorbidities (30%). They were most likely to live in assisted living and least likely to be low‐income. Seventy‐eight percent required assistance with ≥ 2 ADLs, 64% were frail, and virtually all were mobility impaired (99%).

#### 3.2.3 Class 3: Multimorbidity (*n* = 1432, 28.8%)

Members of this subgroup had the highest probability of having ≥ 3 comorbidities (96%), and the lowest probability of dementia (12%). The majority reported dyspnea (56%) and activity‐limiting pain (67%). They had the second highest probability of hospitalization in the past year (46%) and the lowest likelihood of being over 85 (34%).

#### 3.2.4 Class 4: Dementia Plus Multimorbidity (*N* = 1210, 24.4%)

This subgroup was characterized by a high likelihood of multimorbidity (87%) and dementia (84%). Members had the highest probability of having frailty (92%), functional impairment in ≥ 2 ADLs (92%), and hospitalization in the past year (56%). Almost all were mobility impaired, half were low income (51%), and the majority were over age 85 (61%). They had the highest likelihood of having depressive symptoms (67%) and sensory impairment (59%).

### 3.3 Utilization Patterns

The functional independence subgroup had fewer ED visits, hospital admissions, and avoidable ED visits than the total sample and all other subgroups. There were no statistically significant differences in these measures across the other subgroups. The multimorbidity subgroup had more specialty care visits than the total sample and all other subgroups. They also had more primary care visits and combined primary and specialty care visits than the dementia and dementia plus multimorbidity subgroups, with no significant difference for these measures compared to the functional independence subgroup (Table 2).

**TABLE 2:** Health care utilization by latent class.

Utilization measure	Functional independence (*n* = 362)		Dementia (*n* = 629)		Multimorbidity (*n* = 693)		Dementia + multimorbidity (*n* = 518)		Total (*n* = 2002)	
	Weighted mean	95% CI	Weighted mean	95% CI	Weighted mean	95% CI	Weighted mean	95% CI	Weighted mean	95% CI
Any ED visit	36.1	30.2, 41.9	53.9	48.8, 58.9	55.3	49.8, 60.8	55.5	50.0, 61.0	51.7	48.7, 54.6
Any hospital admission	21.0	15.8, 26.2	38.7	33.6, 43.7	42.2	36.9, 47.5	39.8	34.4, 45.1	37.1	34.2, 40.0
Any avoidable admission	5.2	2.7, 7.7	11.1	7.7, 14.5	12.4	8.8, 15.9	13.2	9.7, 16.8	10.9	9.1, 12.8
Any primary care visit	52.1	45.1, 59.2	45.8	40.7, 50.9	62.2	57.2, 67.2	49.4	43.7, 55.2	53.5	50.4, 56.5
Any specialty care visit	54.7	47.9, 61.7	43.2	37.7, 48.7	67.4	62.2, 72.6	47.3	41.5, 53.2	54.7	51.6, 57.8
Any primary or specialty care visit	71.1	64.7, 77.4	60.8	55.6, 66.0	80.2	75.8, 84.7	65.8	60.7, 70.8	70.5	67.8, 73.2

*Note:* Unit of measure, person‐years.

Abbreviation: CI, Confidence interval.

Across all 2202 person‐years, 688 (weighted 29%) observations had no outpatient visits, 836 (weighted 36%) had 1–4 visits, and 678 (weighted 34%) had 5 or more. For observations with 5+ visits, the median number was 8, with an interquartile range of 6 to 11.

### 3.4 Regression Analyses

#### 3.4.1 Level of Outpatient Care and Odds of Any ED Visit

**FIGURE 2:**
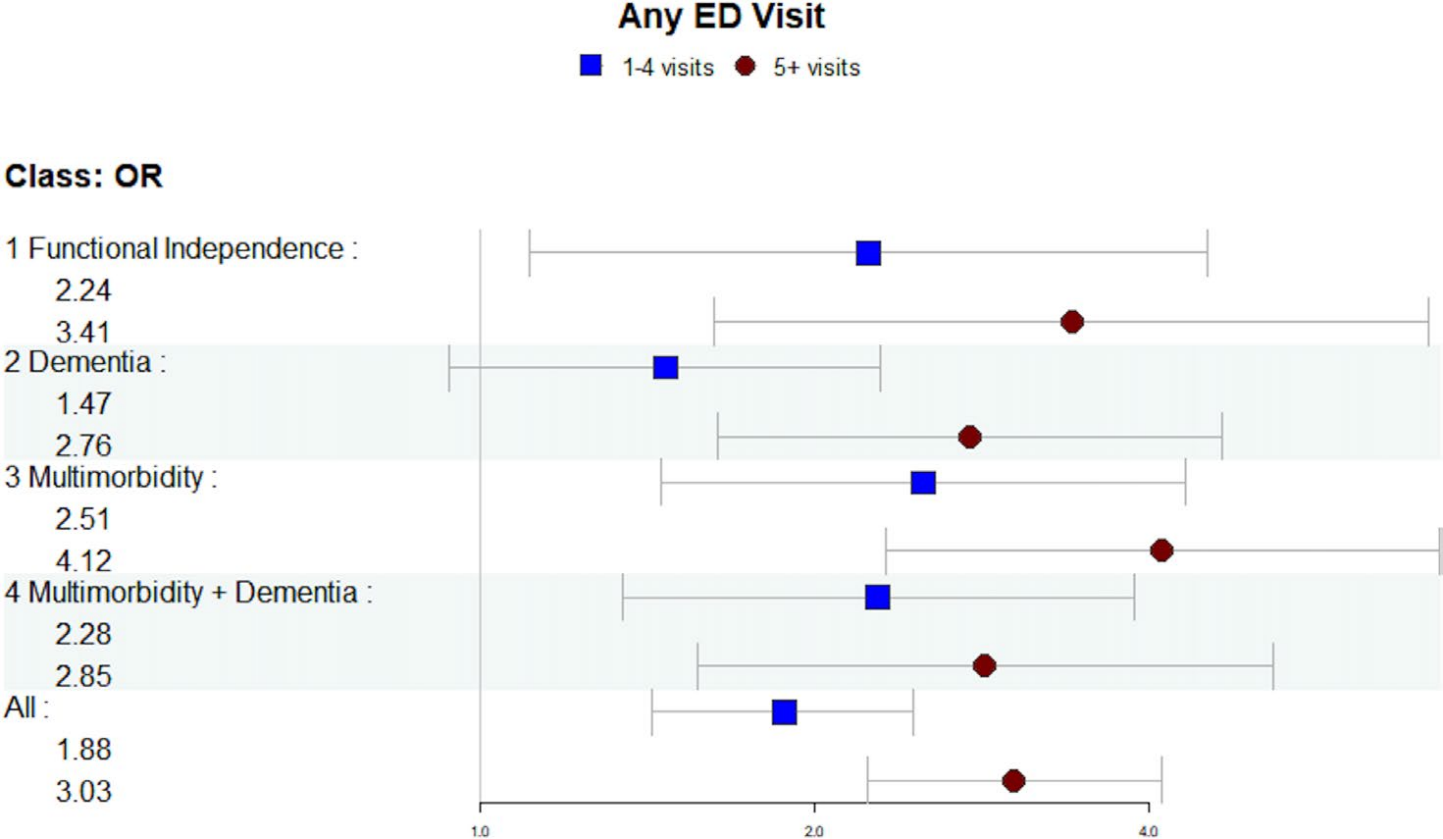
Estimated odds ratios of association between any emergency department visit within 12 months and outpatient care use by latent class and overall sample of the homebound. The reference category is “0 Outpatient Visits within 12 months”. OR, odds ratio. The error bars indicate 95% confidence interval.

Higher levels of outpatient care were associated with greater odds of having any ED visit among the total sample and within each of the four subgroups (Figure 2). Among the total sample, observations with 5+ outpatient visits had three times higher odds of any ED visit (OR = 3.03, CI 2.23–4.11) compared to those with no outpatient visits. In subgroup analyses, the positive association was stronger for the multimorbidity (OR = 4.12, CI 2.23–7.31) and functional independence (3.41, 1.62–7.16) subgroups, and similar for the dementia (2.76, 1.64–4.66) and dementia plus multimorbidity (2.85, 1.57–5.18) subgroups. Across the entire sample and within each subgroup, the odds of any ED visit were higher for observations with 5+ outpatient visits than those with 1–4 visits. Results remained consistent in sensitivity analyses that excluded outpatient visits occurring within 2 and 4 weeks of any ED visit.

#### 3.4.2 Level of Outpatient Care and Odds of ED Visit for ACSCs

**FIGURE 3:**
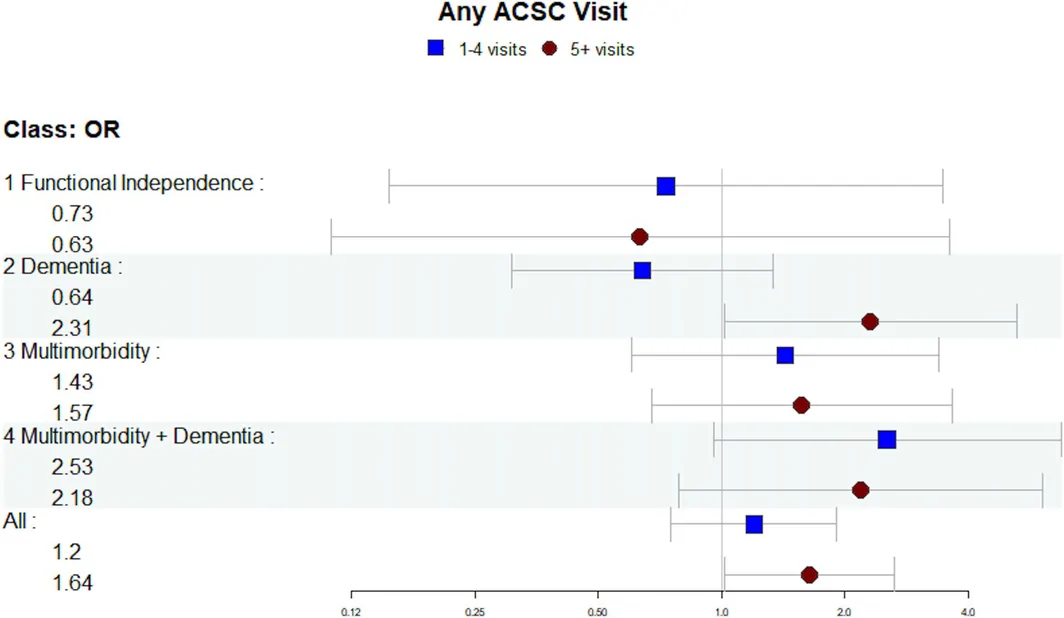
Estimated odds ratios of association between any ambulatory care‐sensitive conditions (ACSC) ED visit within 12 months and outpatient care use by latent class and overall sample of the homebound. The reference category is “0 Outpatient Visits within 12 months”. OR, odds ratio. The error bars indicate 95% confidence interval.

Higher levels of outpatient care were associated with higher odds of any ACSC‐related ED visit among the total sample. Compared to those with no outpatient visits, the 5+ outpatient visit group had more than double the odds of having an ACSC‐related ED visit (OR = 2.23, CI 1.41–3.52). The positive association remained after stratifying by acute (1.64, 1.01–2.64) and chronic (5.99, 2.34–15.28) ACSCs. In subgroup analyses, observations with 5+ outpatient visits had a higher likelihood of any ACSC‐related ED visit among the dementia (2.56, 1.24–5.30) and dementia plus multimorbidity (4.72, 1.87–11.92) classes (Figure 3). There were no significant associations for the functional independence or multimorbidity classes. For acute ACSC‐related ED visits, the dementia subgroup with 5+ outpatient visits had two times higher odds than those with no outpatient care (2.31, 1.01–5.26), while there were no significant associations for the other classes. For chronic ACSC‐related visits, observations in the dementia plus multimorbidity subgroup with 5+ outpatient visits had 30 times higher odds of an ED visit than those with no outpatient care (30.87, 3.50–272.05). However, there was only one observation with a chronic ACSC visit in the reference group, which accounts for the very large confidence interval. There were no significant associations among the other three classes.

## 4 Discussion

This study of a nationally representative sample of homebound Medicare beneficiaries not receiving HBPC makes an important contribution to research on the heterogeneity of the homebound population. The four subgroups identified in our sample of prevalent homebound cases were largely concordant with those identified by Mather et al. in their cohort of incident homebound individuals [6]. While we used a modified nomenclature to characterize the four subgroups, the distributions of indicator variables were similar. Our results further support the hypothesis that distinct clinical and sociodemographic profiles likely shape group‐specific care needs that in turn influence utilization patterns, although we were unable to confirm the conventional wisdom on the interplay between outpatient care and ED use that is associated with being homebound.

To our knowledge, this is the first study to examine utilization among distinct subpopulations of homebound older adults. We found lower levels of ED visits, avoidable ED presentations, and hospital admissions for the functional independence subgroup. This is consistent with the lower burden of chronic disease, frailty, and functional impairment observed for this group. For outpatient care, the multimorbidity subgroup had more specialty care than all other subgroups, reflecting a higher chronic disease burden within this group. The multimorbidity subgroup also received more primary care than the dementia and dementia plus multimorbidity subgroups. These findings may indicate that homebound individuals with dementia face additional barriers to outpatient care beyond what is attributable to multimorbidity, physical impairment and homebound status itself.

Prior studies have shown that older adults with dementia get less evaluation and treatment of comorbid diagnoses and have fewer outpatient specialty visits after dementia diagnosis, while maintaining similar levels of primary care [26, 27, 28, 29, 30, 31]. This results in a net reduction in outpatient care. Possible reasons for these shifts include impaired ability to participate in and/or tolerate treatment or report symptoms that might prompt evaluation; dependence on caregivers for care seeking; potential reluctance among providers to pursue extensive diagnostic workups or targeted treatments in individuals with dementia; and possible interactions between dementia diagnosis and goals of care that may reduce certain types of care seeking. At the same time, older adults with dementia have increased acute care utilization, including more ED visits and hospitalizations [32, 33, 34, 35, 36, 37]. Our results suggest a similar pattern among homebound older adults with dementia. Namely, the dementia and dementia plus multimorbidity subgroups were observed to have less outpatient care than the multimorbidity group, but similar levels of acute care.

In general, homebound older adults use more ED care, but get less outpatient primary and specialty care [9]. A common assumption is that limited access to outpatient care among this group due to physical impairment and transportation barriers leads to increased ED visits, including potentially avoidable visits. We examined associations between levels of outpatient care and ED utilization to see whether an inverse relationship existed for any of the subgroups that would support this hypothesis. We did not find any inverse associations. Several positive associations were observed when adjusting for proxies of medical need (self‐reported health, HCC) and rurality. For the dementia and dementia plus multimorbidity subgroups, higher levels of outpatient care were associated with higher odds of an ACSC (i.e., potentially avoidable) ED visit, though small sample sizes introduced a high degree of uncertainty in effect sizes.

These findings must be interpreted with caution. The cross‐sectional design of this study does not allow for causal inferences, and the covariates we used to adjust for medical need may not adequately capture differences in disease severity or symptom burden that likely drive observed patterns. Furthermore, utilization is not the same as access, as those with the highest outpatient utilization may not be getting as much care as they need, whereas lower outpatient utilization may reflect less care seeking rather than inability to access care. Relatedly, because we did not differentiate outpatient care by setting or quality metrics, we cannot account for how factors such as continuity of care may mediate these effects.

Therefore, the observed positive correlation between outpatient care and ED utilization cannot be interpreted as evidence that barriers to outpatient care do not contribute to higher ED utilization among homebound older adults, or that increased outpatient care directly leads to more ED utilization. At the same time, the finding that homebound individuals who use more outpatient care also use more ED care means that individuals with higher care needs do find ways to access care in different care settings. This suggests that homebound status is not a uniform barrier across individuals. There are degrees of homeboundedness, with the number of individuals in NHATS who never left the home in 30 days being an order of magnitude lower than the homebound population [1]. Homebound status interacts with other mediators of access, differential care needs, and unmeasured complexity. For instance, the positive association between outpatient care and ACSC‐related ED visits among the dementia and dementia with multimorbidity subgroups might reflect differences in goals of care that shape levels of care engagement across settings, including for caregivers. Unmeasured factors such as behavioral complications and caregiver burden may drive increased care seeking in outpatient and ED settings. It is also possible that more outpatient care may generate more ED referrals in some cases, or that more aggressive outpatient treatment of comorbidities may result in more ED presentations for polypharmacy‐related complications. Future work should examine how such factors interact to shape differential utilization patterns.

This study had limitations in addition to those already described. Because we only included Traditional Medicare enrollees, findings may not be generalizable to the Medicare Advantage population [2]. We could not directly compare with those receiving HBPC due to inadequate sample size. Our analysis didn't impose a fixed temporal ordering of outpatient and ED care due to the fluidity of complex care processes. Future research should stratify outpatient care by quality metrics like provider continuity and level of care coordination, which may impact acute care utilization. Similarly, while this study focused on ACSC codes to distinguish appropriate vs. unnecessary care, future analyses may consider other approaches such as disaggregating ED visits by disposition.

In this study of a nationally representative sample of homebound Medicare beneficiaries not receiving HBPC, we found distinct subgroups of homebound individuals, with nearly half having dementia or dementia with multiple comorbidities. While we found significant differences in utilization patterns across subgroups, we were unable to find any evidence that homebound status necessarily connotes an inability to access outpatient care leading to greater ED use.

## Author Contributions

J.L.Y., K.A.O., and B.P.K. conceived the idea, purpose, and design of this article with guidance. J.L.Y. conducted an in‐depth review of the relevant literature. H.C. performed data acquisition, analysis, and visualization. M.L.P. performed project management and coordination. J.L.Y., M.L.P., and H.C. were major contributors in writing the manuscript. K.A.O., B.P.K., and M.L.P. provided methodological expertise, clinical interpretation, and iterative review.

## Funding

MLP was supported by the Epidemiology and Biostatistics of Aging Training Program funded by the National Institute on Aging (grant number T32AG000247) and the Johns Hopkins University Claude D. Pepper Older Americans Independence Center funded by the National Institute on Aging (grant number P30AG021334); KAO was supported by the National Institute on Aging (grant number R01AG060967).

## Conflicts of Interest

The authors declare no conflicts of interest.

## Disclsoure

Authorship has been granted only to those individuals who have contributed significantly to this research and manuscript. All authors provided critical revisions, read, and approved the final manuscript. The research was conducted using secondary data from the National Health and Aging Trends Study (NHATS), which is sponsored by the National Institute on Aging (NIA) (grant number U01AG032947). The funder had no role in the design or conduct of the study; collection, management, analysis, or interpretation of the data; preparation, review, or approval of the manuscript; or decision to submit the manuscript for publication. The authors are grateful to the NHATS study team for providing public access to the data and to the participants for their contributions. The views expressed in this article are those of the authors and do not necessarily reflect those of the NIA or NHATS.
